# Supplementary material for: Genetic interference with HvNotch provides new insights into the role of the Notch-signalling pathway for developmental pattern formation in Hydra
Source: Sci Rep. 2024 Apr 12;14:8553. doi: 10.1038/s41598-024-58837-7 (PMC11014954; doi:10.1038/s41598-024-58837-7)
Supplement: Supplementary file 1 — Supplementary Information. [file 41598_2024_58837_MOESM1_ESM.docx]

**
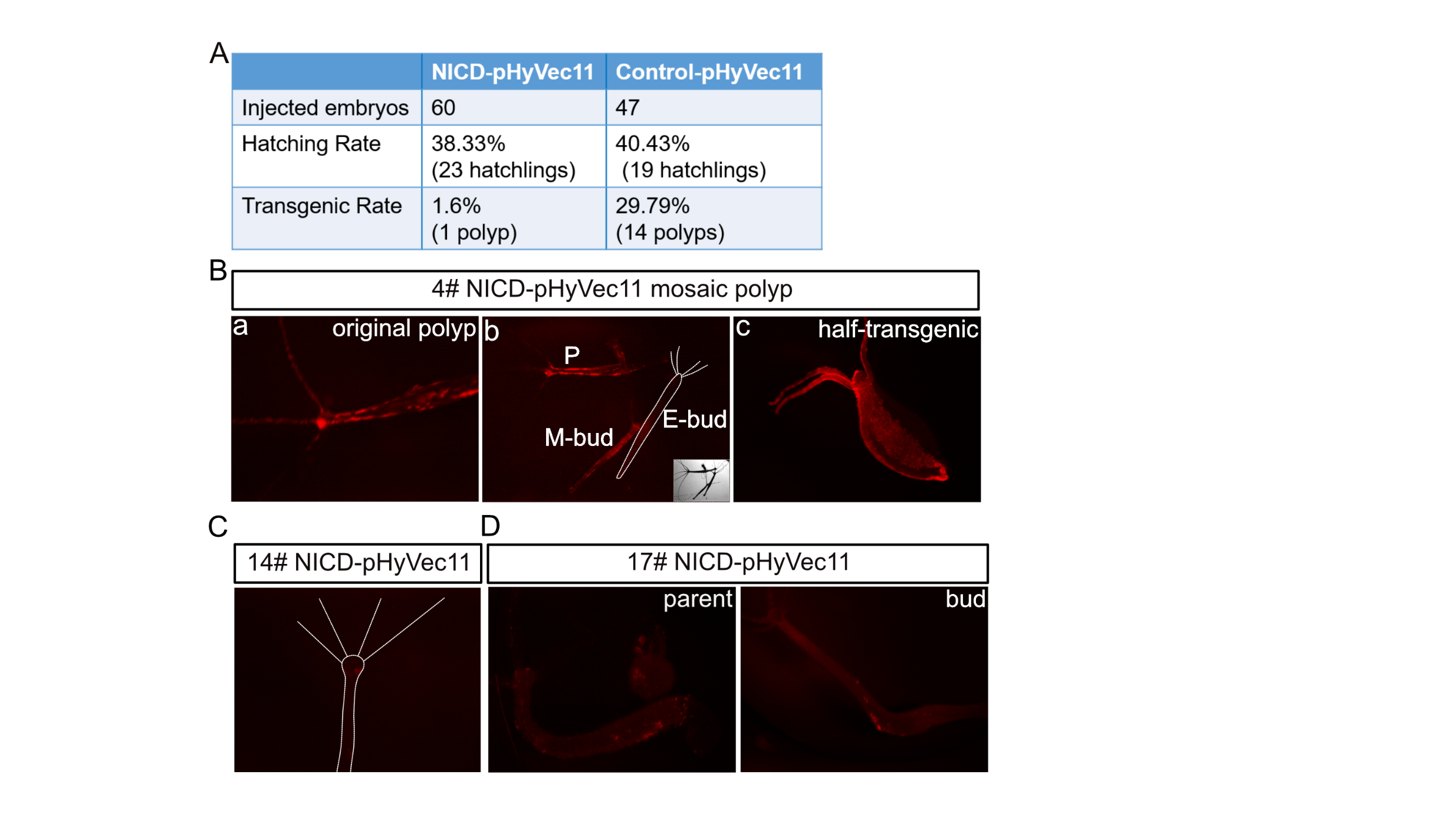
Supplementary Fig. S1 Mosaic HvNICD-overexpressing transgenic *Hydra*.** (A) 60 embryos were injected with HvNICD-pHyVec11, resulting in 23 hatchlings. Strain 4# had most promising DsRed signals. 47 embryos were injected with control-pHyVec11, resulting in 19 hatchlings, 14 of which had good DsRed signals. (B) Polyps from strain 4# with mosaic DsRed fluorescence. (B-a) 4# original polyp. (B-b) 4# original polyp with a developing bud and two detached buds. P: parent polyp; M-bud: mosaic bud; E-bud: empty bud means polyps detached from the mosaic parent, but lacking DsRed signals. E-bud is outlined by dotted lines. (B-c) Half-transgenic polyp obtained from 4# mosaic polyp. (C) Strain 14# injected with HvNICD-pHyVec11 with few signals in the head region, migrating away from the head during development. (D) The original polyp and the first bud from strain 17# injected by HvNICD-pHyVec11, both with small number of scattered DsRed positive cells along the body column.


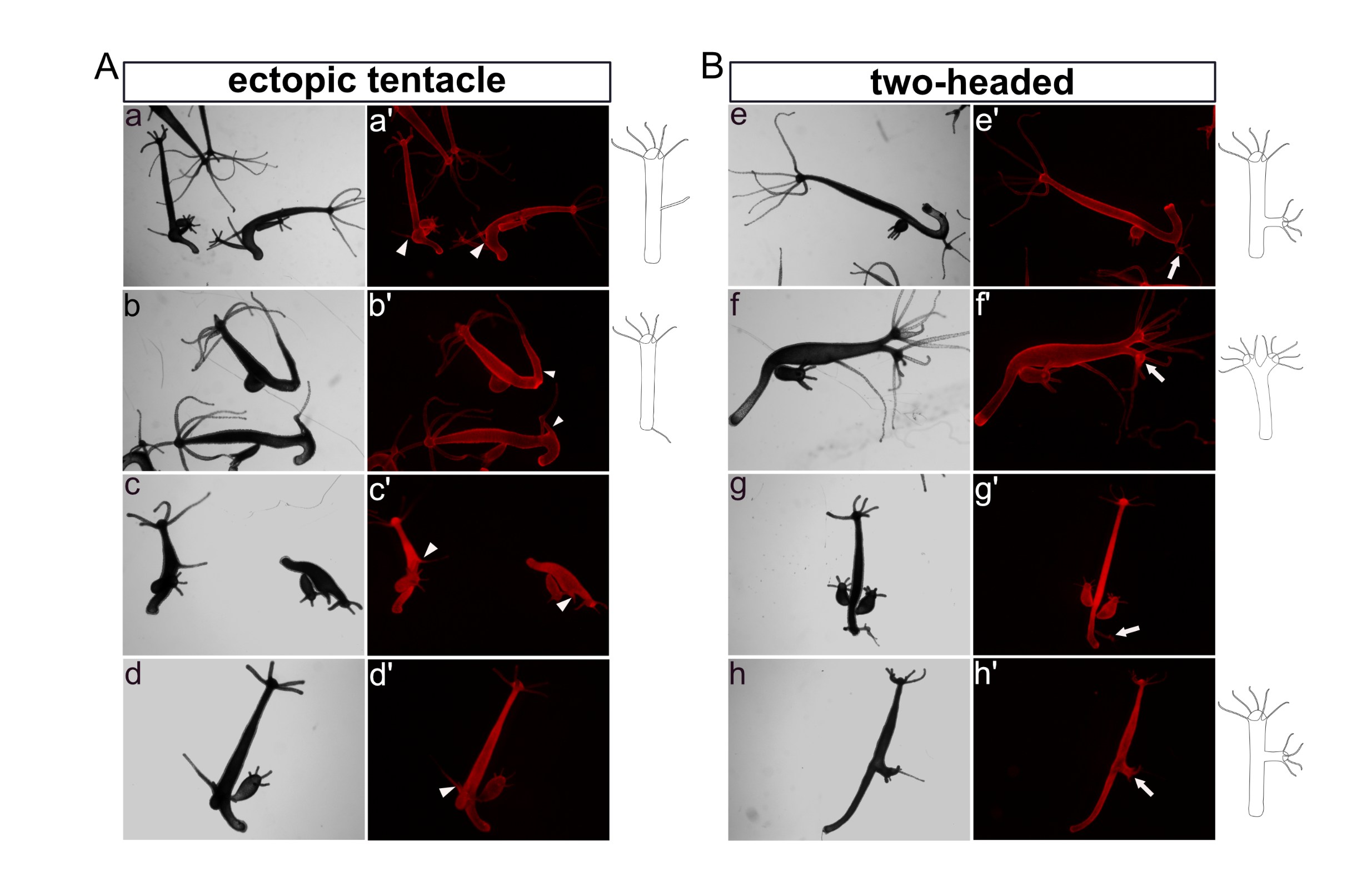


**Supplementary Fig. S2 Phenotypes of HvNICD-overexpressing transgenic *Hydra* maintained for a three-week period.** (A) “Ectopic tentacles”, as indicated by white triangles in the body column (a, b ectoderm-TG; c, d endoderm-TG). (B) “two-headed” phenotype with the extra heads located in different positions along the body column and indicated with white arrows (a, b ectoderm-TG; c, d endoderm-TG).


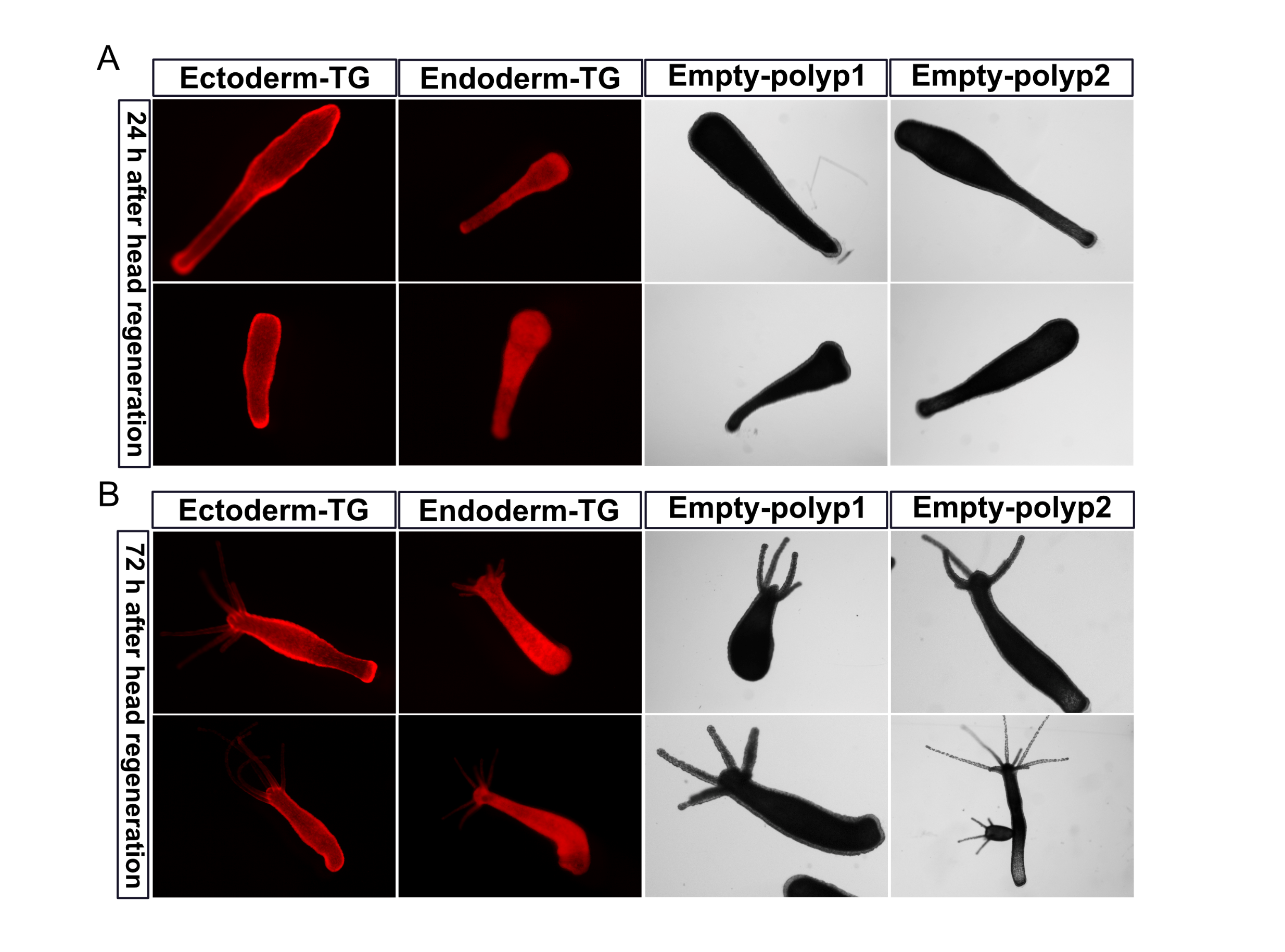
**Supplementary** **Fig. S3 The process of head regeneration in HvNICD-overexpressing transgenic *Hydra* after decapitation below the tentacle ring.** Empty polyps were polyps injected with HvNICD-pHyVec11 but without DsRed signals. (A) The regenerating polyps from the ectoderm-TG, endoderm-TG, empty-polyp1 and empty-polyp2 24 h after decapitation. (B) 72 h after head removal, ectoderm-TG, endoderm-TG and two groups of empty polyps show regular head regeneration.


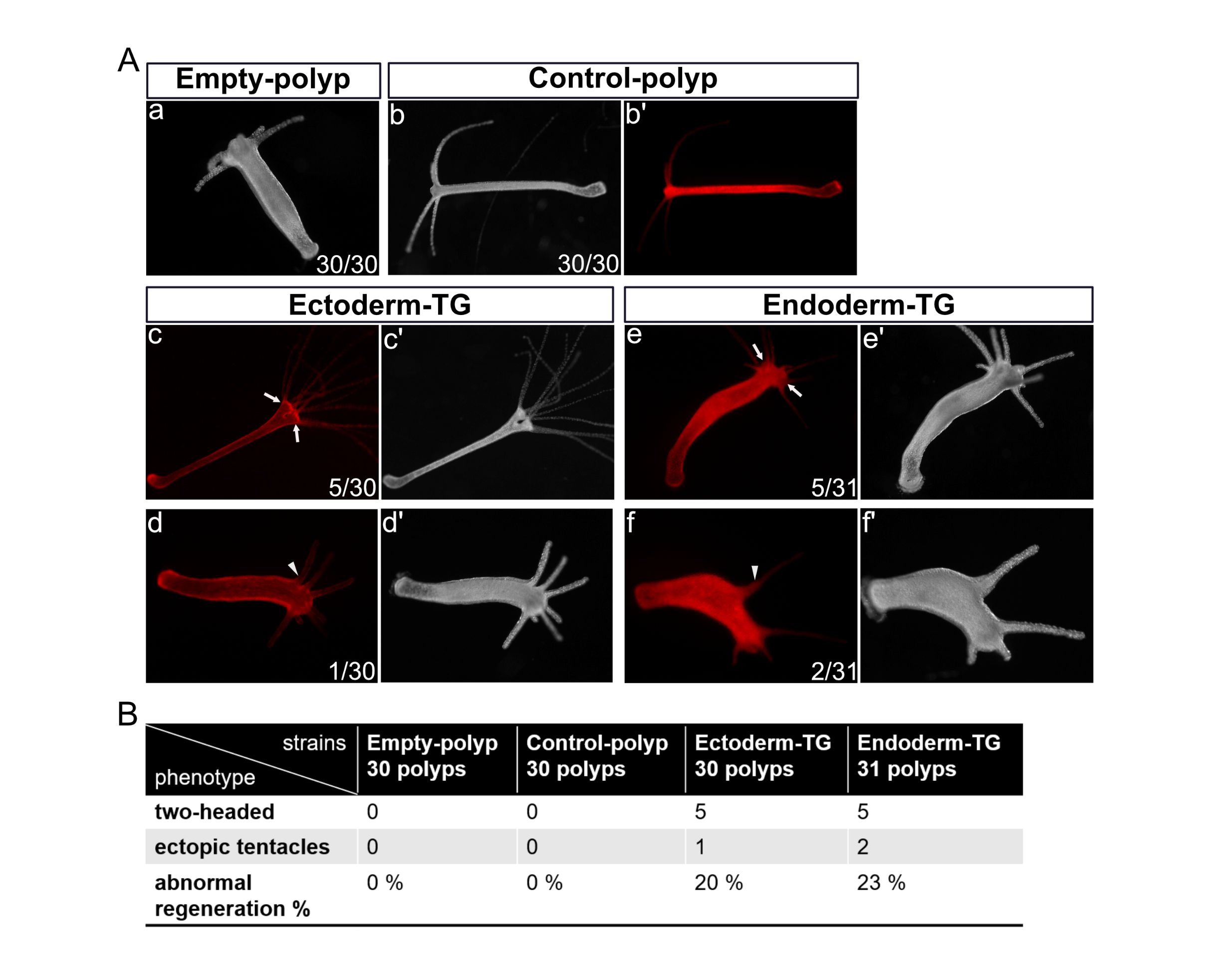
**Supplementary Fig. S4 Head regeneration in HvNICD-overexpressing transgenic *Hydra* after decapitation in the middle of the body column.** Empty-polyp refers to polyps injected with HvNICD-pHyVec11, but lacking DsRed signals. Control polyp refers to polyps injected with the control-pHyVec11 vector. (A) Normal regenerates in empty-polyp and control-polyp, “two-headed” and “ectopic tentacles” regenerates from Ectoderm-TG and Endoderm-TG 3 days after decapitation. Two heads were indicated with white arrows and ectopic tentacles were indicated with white triangles. (B) Quantification of abnormal regeneration percentages in HvNICD-overexpressing and control groups.


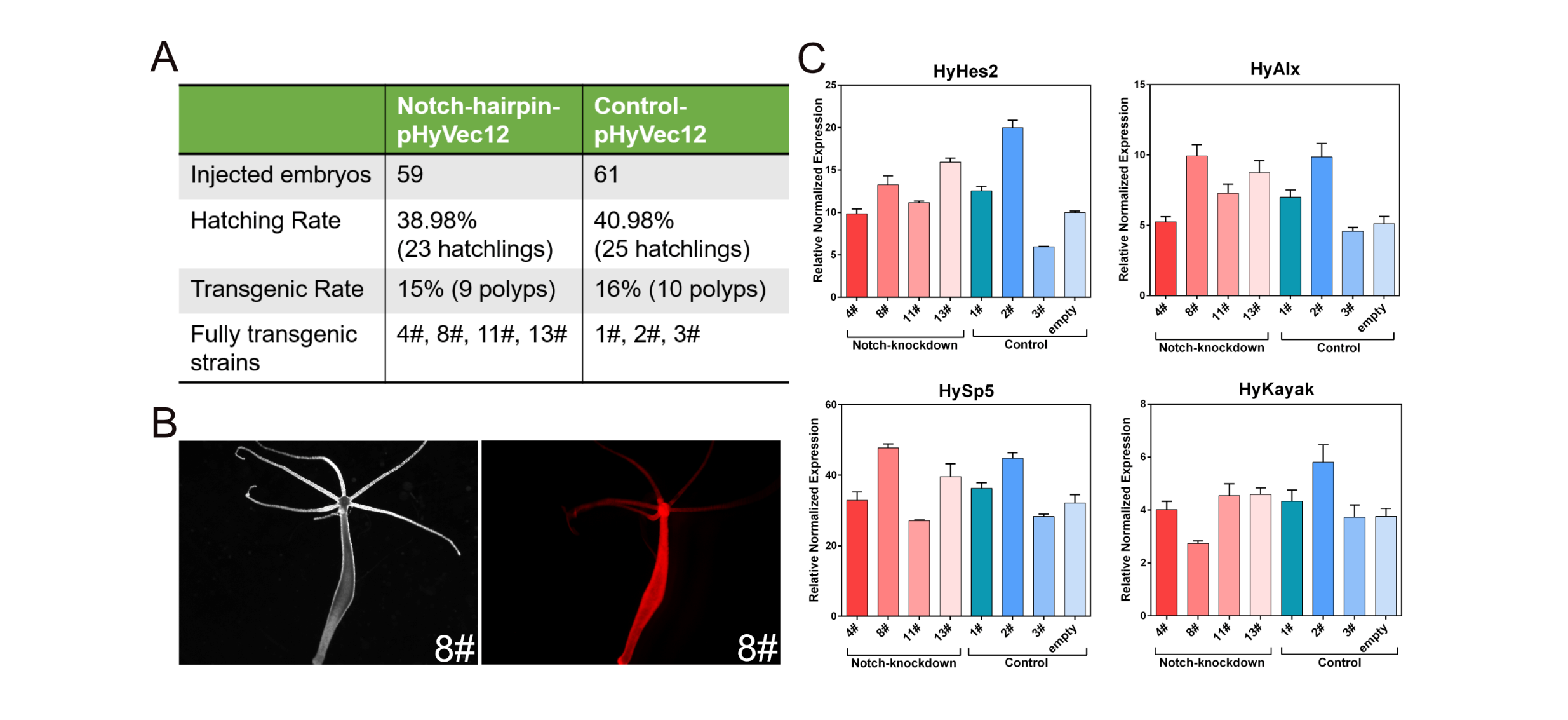
**Supplementary Fig. S5 HvNotch-knockdown transgenic *Hydra* and the expression of HvNotch-target genes.** (A) 59 embryos injected with HvNotch-hairpin-pHyVec12, produced 23 hatchlings. Among them, nine polyps exhibited mosaic DsRed signals. In the end, four strains (4#, 8#, 11# and 13#) with fully transgenic signals were obtained. 61 embryos were injected with control-pHyVec12, resulting in 25 hatchlings and ten polyps with DsRed signals. From this, we generated three fully transgenic control strains (1#, 2# and 3#). (B) Images of fully transgenic *Hydra* from strain 8# expressing DsRed signals in both epithelial layers. (C) Diagram represents the relative normalized expression of HvNotch-target genes after RT-qPCR with mRNA from indicated Notch-knockdown and control polyps. Data for HyHes2, HyAlx, HySp5 and HyKayak are shown, differences are not statistically significant.

**
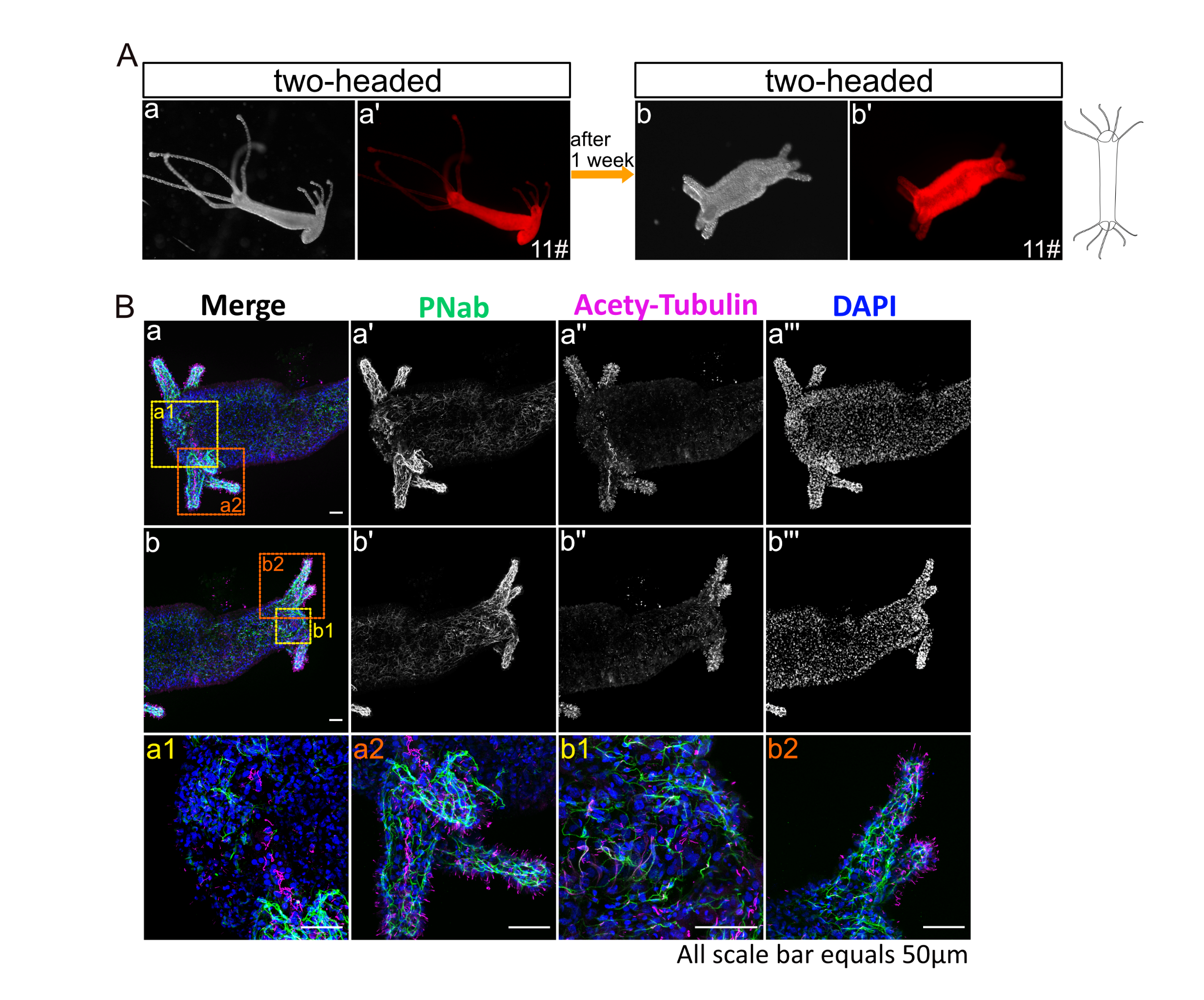
Supplementary Fig. S6 A two-headed polyp from 11# strain of HvNotch-knockdown in the initial stage.** (A) The development of this two-headed polyp with light-microscopy (a, b) and DsRed-fluorescence images (a’, b’) taken at the initial stage and after one-week. (B) Confocal laser scanning microscopic images of this two-headed polyp after co-staining with pan-neuronal antibody (PNab)^1^ (kind gift of Thomas Holstein) to label nerve cells (black and white images a’ and b’), anti-acetylated-tubulin antibodies to label cilia of nematocytes (black and white images a’’ and b’’) and DAPI for staining of DNA (a’’’ and b’’’); merged images of the left head with the enlargements labeled as a1 and a2, b: merged images of the right head with enlargements labeled as b1 and b2).


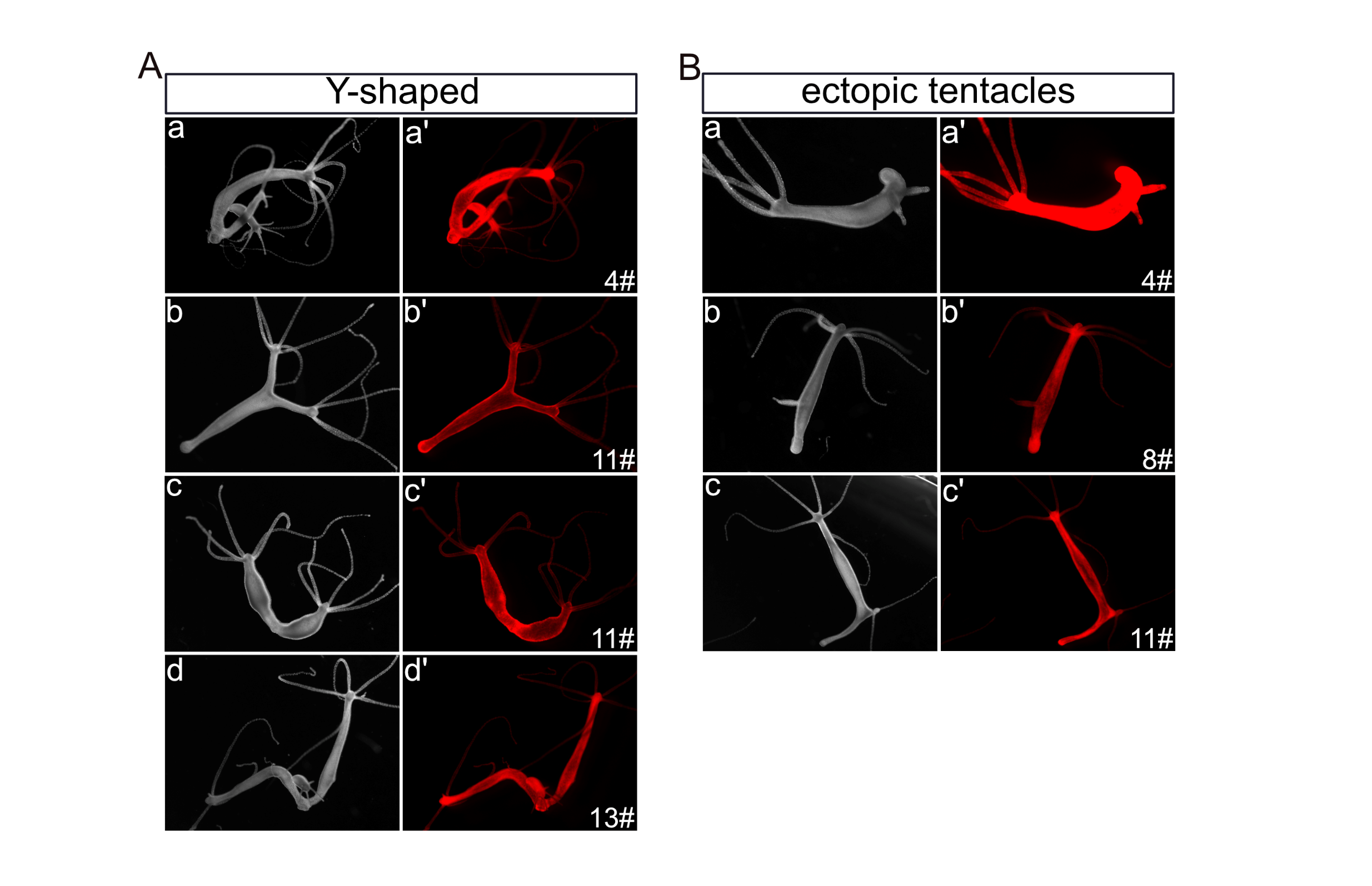
**Supplementary Fig. S7 The phenotypes observed in HvNotch-knockdown transgenic *Hydra* after 6 months**. The images labeled with a-d represent light-microscopy, while a’-d’ display Ds-Red fluorescence. (A) “Y-shaped” polyps in strains 4#, 11# and 13#. Most of joining points located in the foot region (a, c, d) while one positioned in the oral half of the body column (b). (B) Strains 4#, 8# and 11# exhibited “ectopic tentacle” phenotype with one or two ectopic tentacles in the body column.


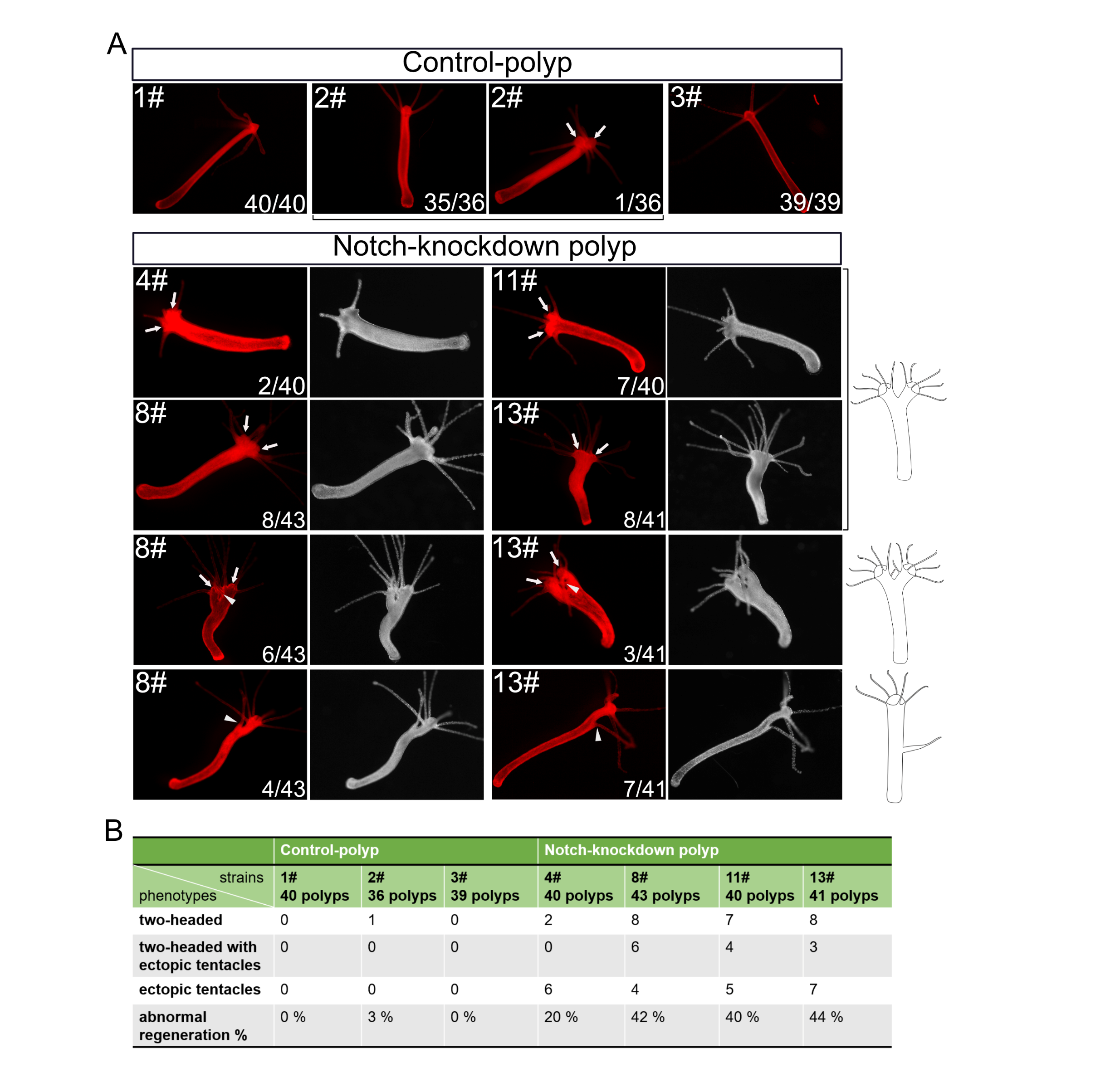
**Supplementary Fig. S8 Head regeneration in HvNotch-knockdown transgenic *Hydra* after decapitation in the middle of the body column.** (A) Images depict the Ds-Red fluorescence and light microscopy of polyps from HvNotch-knockdown strains 4#, 8#, 11# and 13# 3 days after decapitation, compared to control polyps 1#, 2# and 3#. Abnormal regeneration involved “two-headed” and “ectopic tentacles” observed in all Notch-knockdown strains and 2# control strain (1/36). Two heads were indicated with white arrows and ectopic tentacles were indicated with white triangles. (B) Quantification of abnormal regeneration percentage in HvNotch-knockdown and control polyps.


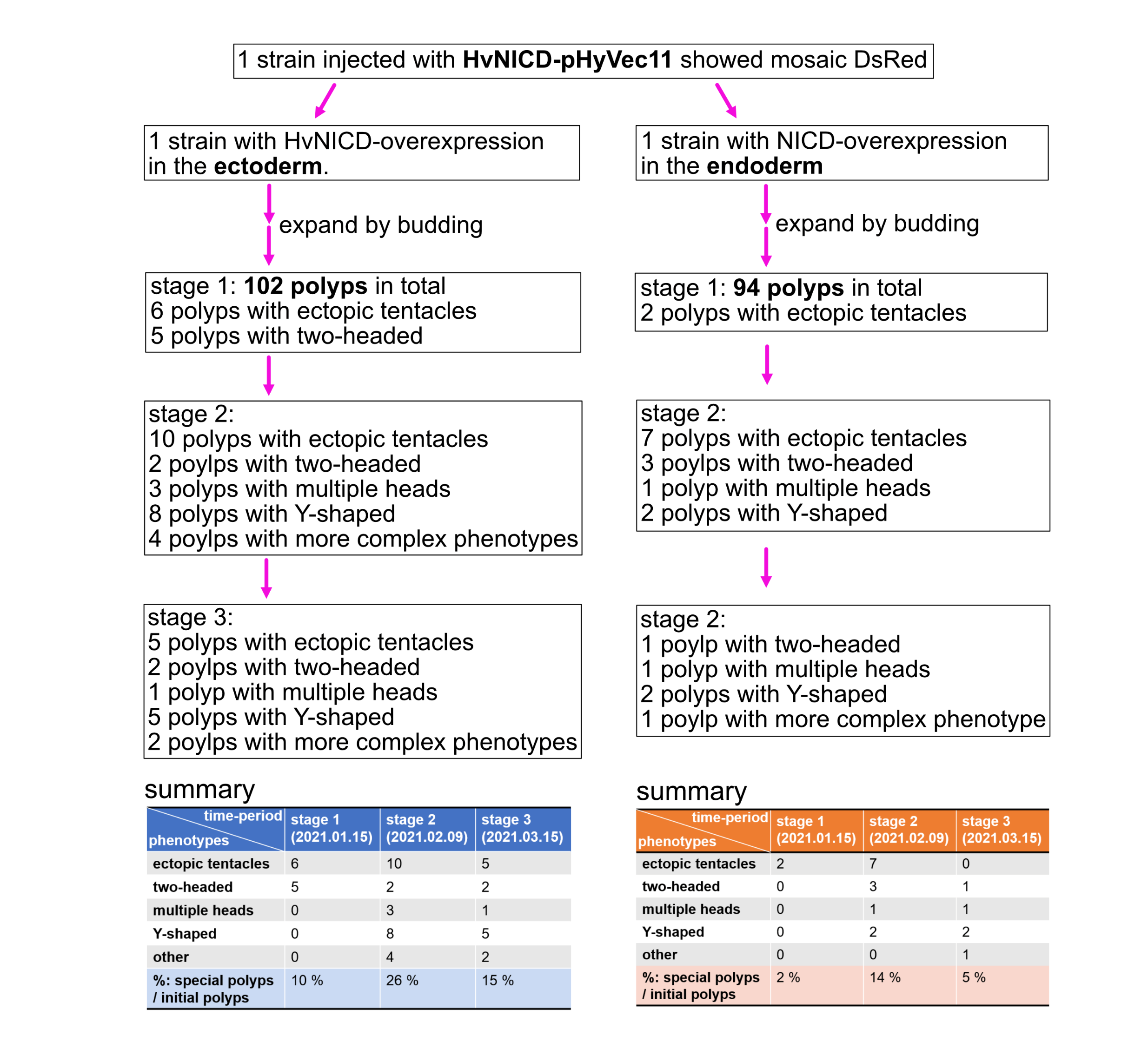
**Supplementary Fig. S9 A summary of observed phenotypes in HvNICD-overexpressing transgenic *Hydra*.** Numbers of polyps with described phenotypes at 3 stages (observation time points) are given for Ectoderm-TGs and Endoderm-TGs. Percentage values was calculated by dividing the number of phenotypes at each stage by the number of fully transgenic polyps initially obtained (ectoderm-TG: 102 polyps or endoderm-TG: 94 polyps).

**
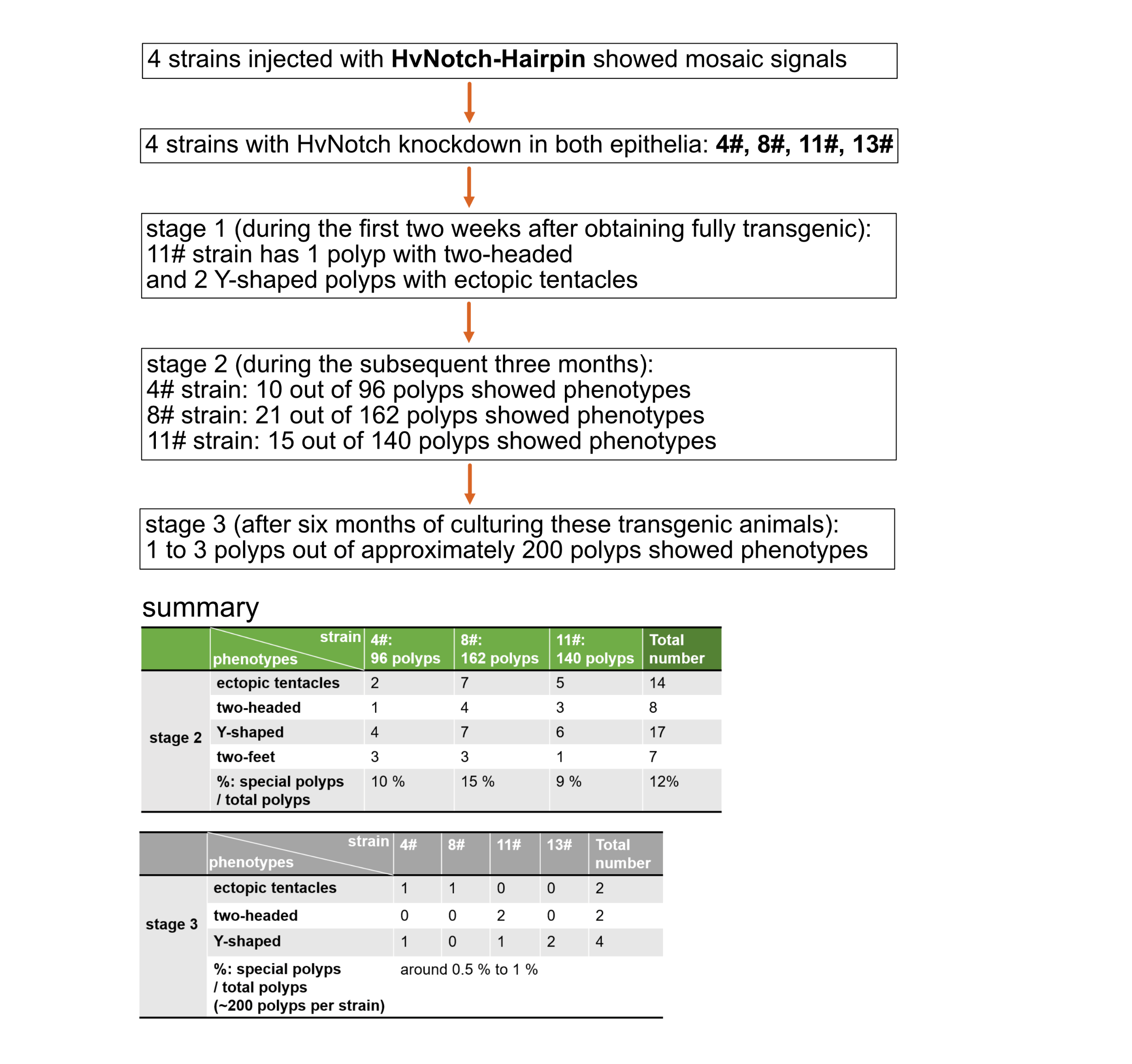
Supplementary Fig. S10 A summary of observed phenotypes in HvNotch-knockdown transgenic *Hydra*.** Numbers of polyps with described phenotypes in strains 4#, 8#, 11# and 13# of HvNotch-knockdown transgenic *Hydra* at indicated stage 2 (approximately three months after obtaining fully transgenic polyps) and stage 3 (after six months).

**
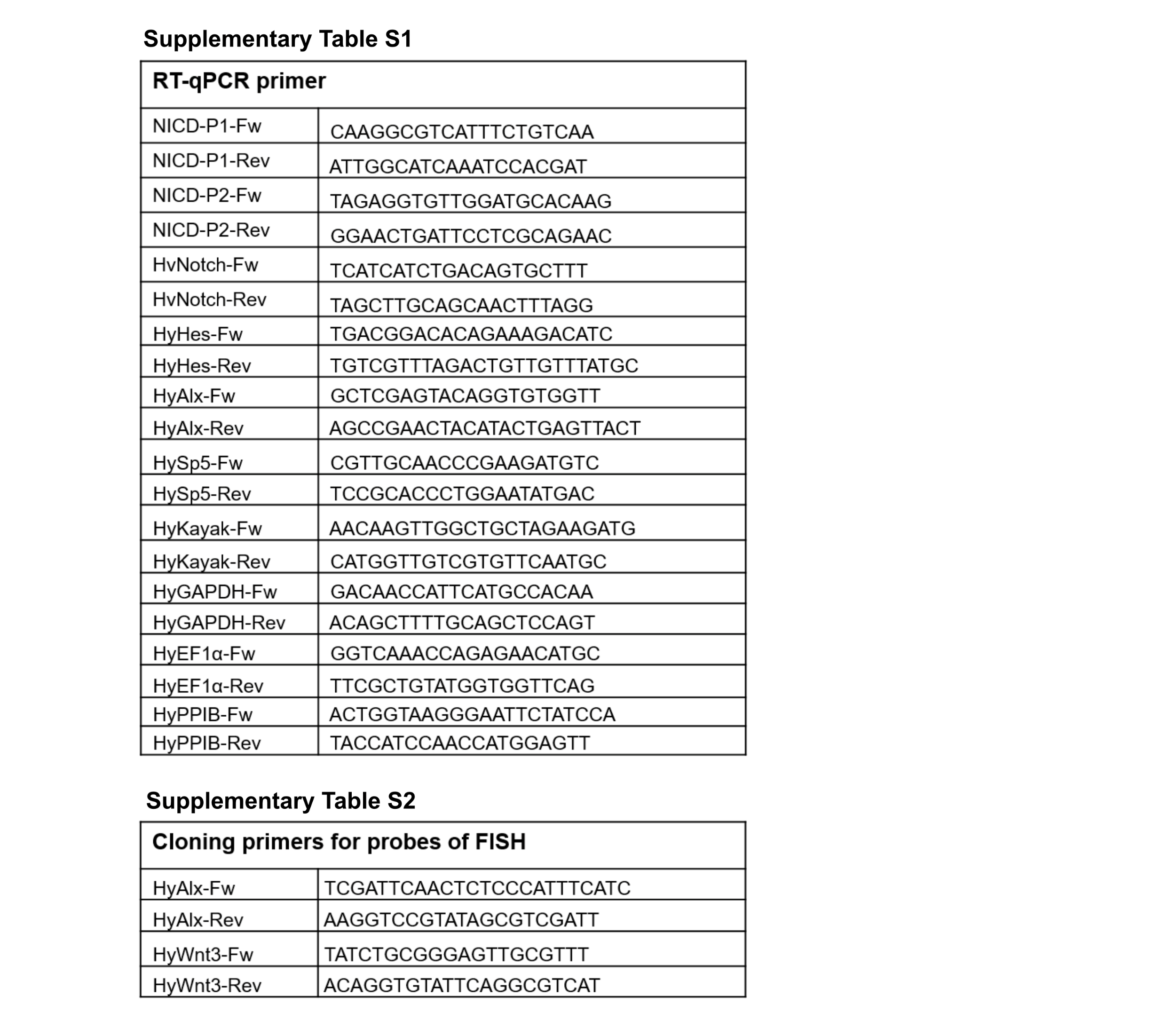
Supplementary Table S1 The list of primer sequences used for RT-qPCR.**

**Supplementary Table S2 The list of primer sequences used to amplify the FISH-probes.**

1 Keramidioti, A. *et al.* A new look at the architecture and dynamics of the Hydra nerve net. *Elife* **12**, doi:10.7554/eLife.87330 (2024).
